# Phosphorene Is the New Graphene in Biomedical Applications

**DOI:** 10.3390/ma12142301

**Published:** 2019-07-18

**Authors:** Marco Tatullo, Fabio Genovese, Elisabetta Aiello, Massimiliano Amantea, Irina Makeeva, Barbara Zavan, Sandro Rengo, Leonzio Fortunato

**Affiliations:** 1 Marrelli Health—Tecnologica Research Institute, Biomedical Section, Street E. Fermi, 88900 Crotone, Italy; 2 Department of Therapeutic Dentistry, I.M. Sechenov First Moscow State Medical University, 119435 Moscow, Russia; 3 Maria Cecilia Hospital, GVM Care & Research, 48033 Cotignola (RA), Italy; 4 Department of Biomedical Sciences, University of Padova, 35100 Padova, Italy; 5 Department of Neurosciences, Reproductive and Odontostomatological Sciences, University of Napoli Federico II, 80131 Naples, Italy; 6 Department of Health Sciences, Magna Graecia University of Catanzaro, 88100 Catanzaro, Italy

**Keywords:** biomaterials, bone tissue, biomedical applications

## Abstract

Nowadays, the research of smart materials is focusing on the allotropics, which have specific characteristics that are useful in several areas, including biomedical applications. In recent years, graphene has revealed interesting antibacterial and physical peculiarities, but it has also shown limitations. Black phosphorus has structural and biochemical properties that make it ideal for biomedical applications: 2D sheets of black phosphorus are called Black Phosphorene (BP), and it could replace graphene in the coming years. BP, similar to other 2D materials, can be used for colorimetric and fluorescent detectors, as well as for biosensing devices. BP also shows high in vivo biodegradability, producing non-toxic agents in the body. This characteristic is promising for pharmacological applications, as well as for scaffold and prosthetic coatings. BP shows low cytotoxicity, thus avoiding the induction of local inflammation or toxicity. As such, BP is a good candidate for different applications in the biomedical sector. Properties such as biocompatibility, biodegradability, and biosafety are essential for use in medicine. In this review, we have exploited all such aspects, also comparing BP with other similar materials, such as the well-known graphene.

## 1. Introduction

Modern biomaterials must meet several requirements, even involving their biological and structural characteristics. Recently, scientists have taken smart two-dimensional materials (2D materials) into consideration, as the allotropic forms of many such materials have shown peculiar characteristics which may be usable in several applications. The interface between cells and biomaterials should be biocompatible and bioactive: 2D materials are tunable on the nanometric-scale, which can be used to improve the connection between such materials and human tissue. 2D materials have consistently shown unique physical, chemical, electronic, and optical characteristics; recently, MoS_2_, WSe_2_ and h-BN have been shown to be useful in the production of many biomedical devices. In this context, graphene was recently investigated for its properties which show great potential for use in biomedical applications [1,2]. Graphene has physical, antibacterial, and electrical peculiarities that make it ideal for biosensors and medical devices; however, it also has limitations—such as biodegradability in vivo and the cytotoxicity in vitro—that have pushed scientists to search for alternative solutions.

Among the allotropes of phosphorus which are already present in different percentages in our organism, black phosphorus has structural properties which are interesting to investigate in the light of future applications in the medical sciences. As previously reported, other materials are currently under evaluation for use and integration in future medical devices. MoS_2_, WSe_2,_ and h-BN appear to be biocompatible, similarly to graphene and Black Phosphorene (BP), but with evident differences, especially in their electronic performance. H-BN is an insulator, and recent studies on it have mainly focused on its thermal conductivity and ability to transport phonons, for use in the construction of fuel cells that can also be adopted in the biomedical sector. High electrical resistance does not facilitate use in applications for electrochemical sensors or wearable devices, in which the detection of an electrical signal is fundamental [3]. MoS_2_ and WSe_2_, as reported by Akinwande et al. [4] and Sahoo et al. [5], have shown peculiar characteristics in the ON-OFF current ratio, which may be useful for the realization of Field Effect Transistor (FET) sensing, even if the low band-gap (1.2–1.8 eV) limits their use in some fields of biological analysis. 2D nanosheets of black phosphorus are called Black Phosphorene (BP) [6]. The structural anisotropy of BP contributes to optimizing its mechanical, optical, electrical, and thermoelectric conductivity properties for various applications. Briefly, the chemical structure of BP consists of a phosphorus atom covalently linked with three adjacent phosphorus atoms, which generates a crystalline structure characterized by a hexagonal shape [7]. In recent years, several studies have been carried out to evaluate the optoelectronic, photothermic, photodynamic, and electrochemical behavior of BP. It is important to consider that nanomaterials, though biocompatible, may induce inflammatory responses that are not easy to control and heal. This issue should be carefully evaluated before any biomedical application of these nanomaterials. BP is already a bone constituent, albeit in small percentages, constituting −1% of total body weight (about 660 g on average) [8]. In tissue engineering, it is well known that calcium and phosphates play an important role in bone regeneration. Regarding nanomaterials made by 2D layers of such components, the concern is mainly related to the amount of in situ nanoparticles which are released, and their long-time toxicity. Currently, BP seems to be safe due to its chemical and molecular properties that ensure high stability and biocompatibility [9]. These properties may allow BP to be used in various applications, ranging from biosensors (electronic, colorimetric, fluorescent, electrochemical) to medical imaging, from pharmacological applications to serving as a coating on scaffolds and prosthesis surfaces. This review outlines recent trends and future insights of biomedical applications based on BP, paying specific attention to the following: (i) the physicochemical properties of BP, (ii) the biological properties of BP, (iii) BP synthesis and production, (iv) biomedical applications of BP, and (v) presenting some promising insights on the future applications of BP in biomedicine.

## 2. Physicochemical Properties of BP

### Structure of BP

Phosphorus represents about 1% of the total human body mass, being found specifically in bones. The chemical structure of phosphorus allows it to create links with any other atom by hybridization of its orbitals, obtaining an sp^3^ form.

The BP presents an anisotropic reticular structure, which generates two atomic layers with two different interatomic bonds. In this regard, under high pressure, the crystalline structure of BP can be arranged into two different configurations. The structure, under controlled thermal conditions, can shift from an orthorhombic to a rhomboid form at a pressure of 5.5 GPa. Another transition can be obtained after the application of a pressure of 10 GPa, i.e., the rhomboid configuration shifts to a cubic one. Among the different BP tridimensional conformations, the BPQD (BP Quantum Dose) has been one of the most investigated in the recent literature; it has an orthorhombic crystalline structure, which, based on the type of synthesis process, makes it possible to obtain different isoforms that are capable of changing according to mechanical stresses and the intended use. For example, BPQDs maintain a certain structural stability when used in applications aimed at drug-delivery and bio-imaging. In this context, BPQD has demonstrated that its high stability makes it possible to improve its properties related to light absorption. Moreover, BPQD is able to fluoresce in a way that makes it useful in the diagnosis of cancer. The cubic crystalline configuration, on the other hand, is obtained at high pressures, and allows a redistribution to occur of the electronic density that favors phosphorene conductivity [6]: In this conformation, the use of the BP in medical devices can be considered, in which the sensitivity of electronic elements is essential [4,5]. Given the possibility of obtaining different structural configurations, future studies could concentrate on the definition of the factors that influence the aforementioned transition, in order to obtain adequate characteristics for the chosen field of application, such as biosensors, polymeric scaffolds and smart drug-delivery systems [10].

**Figure 1:**
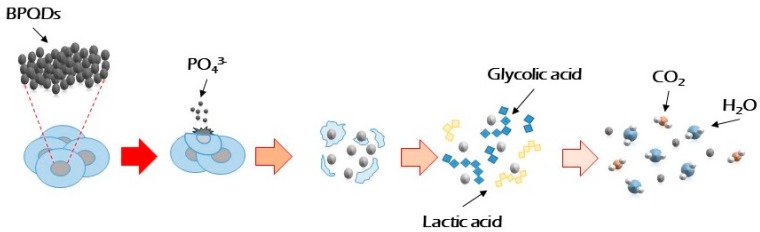
Process of degradation of polymeric scaffold (PLGA: poly lactic-co-glycolic acid), mixed with BPQDs in a physiological environment. Adapted from Reference [13].

Some features make the BP unique, compared to graphene and other biomaterials. In fact, BP is among the most stable biomaterials at room temperature and under normal pressures. It can degrade easily and generate rapid fluorescence in some conditions [11]. Although its use for electronic sectors is not recommended, BP is a perfect fit for the medical sector, mainly thanks to its high biocompatibility and biomimetic biodegradability that reveal its potential in, e.g., bone repairing. Moreover, the excellent optical properties of BP are ensured by a good absorption coefficient in visible, infrared (IR) and ultraviolet (UV) light. In contrast to graphene and other 2D materials, this allows BP to be used for colorimetric and fluorescent detectors, as well as for biosensing devices. For example, Zhao et al. have shown that the BP makes it possible to detect precise analytes (such as immunoglobulin IgG, myoglobin Mb) and inorganic ions, highlighting the photodynamic and electrochemical properties that make it unique [12]. A physical peculiarity, which is present also in other 2D materials such as graphene or borophene, is related to BP’s anisotropic properties; interestingly, there are several techniques by which to synthesize BP, depending on the final application [13] (Figure 1).

An important evaluation regarding the advantages of BP, compared to graphene and other 2D materials, such as MoS_2_ and WS_2_, is related to its lower cytotoxicity, higher in vivo biodegradability, and its tendency to release few nanoparticles in the human body [14]. The toxicity of 2D materials can be influenced by physical parameters, such as size, distribution, concentration, and shape, but also by the time of their exposure to biological tissues. In particular, the toxicity of graphene-based nanomaterials has been mainly linked to several oxidative pathways, and to biological damage involving cell membranes, resulting from the direct interaction between graphene and cells. In this light, studies were carried out on hMSC, human erythrocytes, skin fibroblasts, and glioblastoma cells. It has been hypothesized that the mechanism underlying the cytotoxic process, which basically depends on the concentration of graphene, is linked to the activity of the Reactive Oxygen Species (ROS) generated by graphene, as well as to the direct interaction between graphene and membrane phospholipids [15]. The in vitro cytotoxicity of MOS_2_ and WS_2_ was shown to be much lower than that of graphene, as the cells maintained their vitality even when they were exposed to concentrations as high as 100μg/mL [16]. Specific studies on the viability of cells in vitro showed unprecedentedly low, or a total absence of, cytotoxicity at 1.0 mg ML of BP [17].

**Figure 2:**
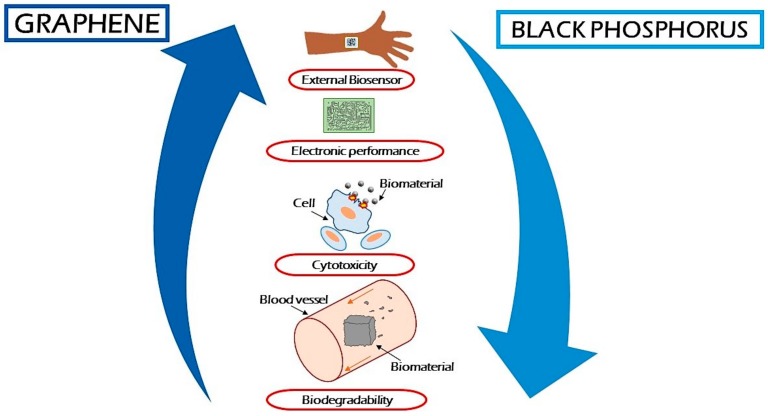
Main applications of Black Phosphorous (BP) compared to graphene in biomedical fields.

The BP monolayers, due to their zig-zag conformation, do not excel in terms of Young’s Modulus and thermal conductivity, compared to graphene. On the other hand, they have higher optical absorption and a lower presence of impurities, which facilitate their use for optical pulse detection devices [12]. Two-dimensional materials such as graphene, have good electronic mobility but a fairly low “ON-OFF” current ratio that could impede their use in sensing applications. BP is highly sensitive to electrical disturbances, a characteristic which facilitates its application for gas detection devices [18]. The optical properties of BP are strongly linked to its band-gap; the band-gap of BP nanosheets can be easily modulated from 0 to about 1.45eV through the application of external stimulus. The control of the band gap is fundamental for the use of BP in photothermic, photodynamic, biosensing, and bioimaging applications [19] (Figure 2).

## 3. Biological Properties of BP

In the ongoing search for ideal 2D materials for use in biomedicine, a key factor is biocompatibility. In different studies, graphene and BP have been reported to be biocompatible. In detail, BP showed fewer inflammatory reactions, lower cytotoxicity at high concentrations, and better control of corrosion in vivo. All such properties are required for biomedical applications. Moreover, BP can also be used as a coating for the surface of other materials, thus creating composed bilayered scaffolds which are characterized by high chemical and structural stability, and by a low corrosion curve in biological environments [20]. In contrast, the interaction between BP sheets and molecular oxygen seems to worsen the structural properties of this nanomaterial; in fact, oxygen can link the atoms of phosphorus by covalent bonds, thus increasing the overall degradation rate during their interactions.

Moreover, O_2_ easily dissociates from the BP surface, thereby creating an oxidized covering. Water could also induce structural changes in BP sheets; in fact, the naïve BP hydrophilicity has a strong impact on the degradation rate [21]. In this regard, we may also consider using the influence of oxygen and water on the BP degradation rate to our advantage, as a high degradation rate may be required in some therapeutic or pharmacological applications.

## 4. BP Synthesis and Production

To produce nanosheets of 2D materials, we will use two different approaches: the “Top-down” and the “Bottom-up” method. The top-down method consists of the realization of a single layer through different exfoliation techniques (mechanical, liquid, and chemical). On the other hand, the bottom-up method involves specific chemical reactions (chemical wetting, CVD chemical vapor deposition) to compose the layer. Both such techniques have advantages and disadvantages. In the top-down approach, nanosheets can have several sizes, but the chemical residues generated by the various exfoliation techniques remain. The bottom-up technique makes it possible to generate BP layers chemically with more compact structures; moreover, it can be used to combine more materials together. On the other hand, this technique showed a low ability to modulate the width and thickness of the single layer [22]. Now we will describe the main synthesis techniques for BP sheets.

### 4.1. Mechanical Synthesis 

This technique is used to realize nanosheets with a fairly large surface/volume ratio. Adhesive tapes are capable of detaching thin strips of BP, which are then returned to the SiO_2_-based substrate. To eliminate residues released by the adhesive, BP strips are heated up to 180 °C. Although this technique guarantees the synthesis of highly pure BP nanosheets, currently, it is not seen as optimal; in fact, BP produced with this technique does not necessarily demonstrate the same thicknesses of layers. Alternative techniques have been investigated using polymers synthesized and formed at specific temperatures [23].

### 4.2. Liquid-Based Synthesis

This type of exfoliation requires the use of ultrasound technologies; it further requires immersion in solvents (such as chloroform, ethanol, tetrahydrofuran, and other halides). To obtain optimal BP nanosheets with this technique, one must a process where temperature (in some cases, around 140 °C) and time (h) are two critical factors. Generally, the BP is placed inside a solution with 1-methyl-2-pyrrolidone as a solvent (the method is based on the passivation of the surface); then, it is exposed to ultrasounds for up to six hours, centrifuged, and finally, dispersed in water [13]. The issue is related to the need to avoid direct contact between BP and oxygen or water as much as possible. To overcome this issue, Wang et al. [24] proposed the use of argon to remove the oxygen molecules present in the solution; this would inhibit the degradation of BP.

### 4.3. Electrochemical Synthesis

The electrochemical technique involves the use of a platinum electrode. A slight voltage is applied between the platinum and BP, so as to generate a flow of free radicals. Because of the resulting oxidative process, this technique can produce thin layers of BP [18]. As previously stated, oxygen favors a quicker degradation of BP; accordingly, it impacts on the overall stability of the crystalline structure, promoting rapid superficial corrosion of BP sheets.

### 4.4. Plasma-Based Synthesis

This technique can be combined with those based on mechanical exfoliation to minimize the aforementioned problems. Plasma engraving obtained with Ar^+^ gas glow discharge ensures considerable advantages, notably: (i) the reduction of all impurities typically on the adhesive tape; and (ii), this technique can lead to better precision of the proportions of the BP layers. This is possible thanks to the modulation of engraving times, which would allow us to better control the production stage [25].

### 4.5. CVT (Chemical Vapor Transport) Synthesis 

Among the bottom-up techniques, CVT is the one which shows the most promise in the synthesis of BP. This technique needs a chemical reaction to occur between a solid and a gas, resulting in the formation of mono-layers of BP after precipitation [26]. More in detail, the molecules of BP are used as precursors within a tube filled with argon. Once both temperature and time have been set, there is the further step: deposition. To remove impurities, several baths in toluene or acetone are suggested. Therefore, as previously described, numerous production techniques will produce BP nanosheets which are characterized by different and specific chemical-physical properties. Currently, Raman spectroscopy is used to evaluate the purity of the BP samples, using a laser operating at 514 nm. Then, using techniques such as HRTEM (high-resolution electron transmission microscopy), AFM (atomic force microscopy), and XPS (X-ray photonic spectroscopy), the structures of BP can be investigated at high resolution [22].

## 5. Biomedical Applications of BP

### 5.1. Biosensors

**Figure 3:**
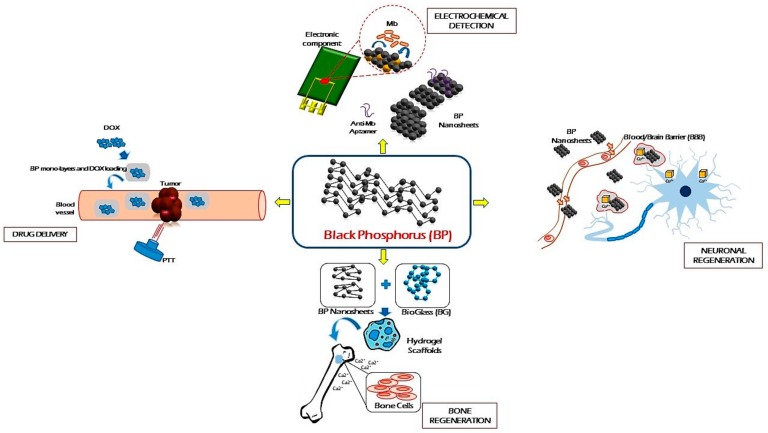
Strategic applications of Black Phosphorene (BP) in biomedical fields. Adapted from Reference [9].

BP is a good candidate for several strategic applications in the biomedical sector (Figure 3). Properties such as biocompatibility, biodegradability, and biosafety are essential for use in medicine. As will be further described, recent studies on BP have shown it to be among the 2D materials which are applicable in more than one biomedical field. Its use has been considered in the therapeutic, in imaging, pharmacological, and diagnostic fields, as well as in the realization of biosensors, and in bio-printing. Wang et al. highlighted the excellent optical properties of BP in phototherapy. In fact, BP can be used as nanoagent in vivo, which, during irradiation with NIR light, can selectively kill tumor cells within a well-defined time (typically, 8–10 min). As confirmation of recent in vitro studies reporting interesting proof of its use in oncology, the behavior of BP in oncological phototherapy showed an efficiency higher than those of other traditional photothermic agents. Another application is related to photoacoustic imaging, in which BP was combined with TiL_4_ to be used as an exogenous agent. The TiL_4_-BP combination has made it possible to obtain better stability in water, avoiding degradation issues [27]. Also, BP has been shown to have electrochemical, fluorescent, and electrical properties, which may make it suitable for use in biosensors-related technologies. Many applications in the clinical field involve the use of technologies which are able to define the concentrations of certain substances in biological fluids, e.g., in human blood. BP nanosheets and nanoparticles are often used in the realization of sensors inside analytical instruments. Typically, a biosensor made of BP is used for the detection of immunoglobulins, cardiac markers, and early oncological markers [13]. In 2016, Sofer et al., studying the properties of BP nanosheets, reported excellent results after combining gold nanoparticles with BP sheets. The presence of BP increased the already excellent conductivity of gold, also increasing its sensitivity in detecting poorly circulating biomarkers [28]. Many sensors for medical applications can also detect several substances in healthcare environments. The BPQD (BP Quantum Dose) is used for devices which are able to detect humidity in medical equipment, such as sterilizers, incubators, and surgical instruments [13].

### 5.2. Bioscavenger

In neurodegenerative disorders (ND) such as Parkinson’s, Huntington’s, and Alzheimer’s disease, the peculiar homeostasis of Cu^2+^ can lead to neural cytotoxicity [29]. The high incidence of these pathologies has led researchers to consider experimental treatments which are increasingly in line with modern medicine [30].

Other studies have been focused on 2D nanomaterials which are capable both of crossing the blood-brain barrier (BBB) without any side effects, and of reducing metal oxidation.

In 2018, a study by Chen et al. demonstrated how BP nanosheets can effectively capture Cu^2+^, thus protecting neural cells from cytotoxicity. BP 2D nanosheets, tested both in vitro and in vivo, have shown exceptional clinical properties when used in subjects affected by ND. In fact, BP has shown the ability to selectively capture Cu^2+^, among the various metal ions present in the organism (such as Mg^2+^, Fe^2+^, Fe^3+,^ and so on). Excellent results were also obtained in photothermic therapy, in which BP biofilms guaranteed excellent permeability of the BBB, improving the pharmacological efficacy and minimizing the problems of chemical cross-reactions among the various drugs used in treatments [31].

### 5.3. Medical Imaging 

The structural stability and optical properties of BP make it an ideal candidate for therapeutic and diagnostic applications in oncology. The nanolayers of BP are used as carriers of targeted drugs. In photothermic therapy, BP has been demonstrated to have excellent properties related to the absorption of light: when it accumulates on the tumor mass, thanks to the use of surgical lasers, the warmth will quickly destroy the tumor mass. Even in medical imaging, the behavior of BP is very promising; in fact, when it is absorbed by the tumor mass, it generates a fluorescence which may serve to define the morphology of cancer with extraordinary precision. Finally, it may also be used for photoacoustic imaging [32]. Through liquid exfoliation, we can obtain nanosheets of BPQDs that, as previously mentioned, maintain BP’s performance in diagnostic applications [33]. During synthesis by exfoliation, BPQDs can be combined with TiL_4_, at a specific temperature and time (approximately 15 h). This combination generates TiL_4_@BPQDs, interesting nanosheets which may be exploited as a contrast agent for photoacoustic imaging (PAI), a technique adopted in vivo, which has shown remarkable success in terms of PA response under near-infra-red (NIR) stress. TiL_4_@ BPQDs has excellent potential in diagnostic applications because of its excellent sensitivity and high spatial resolution in detecting tumor masses [34].

### 5.4. Scaffolds and Coatings

BP is an ideal candidate both as a coating and as a transporter. Wei Tao et al. highlighted the efficacy of BP nanolayers coated with PEG (polyethylene glycol) to administrate Doxorubicin (DOX) in oncological chemotherapy. More in detail, BP nanosheets loaded with DOX can selectively degrade in the target area with better efficacy. Moreover, thanks to the photodynamic, photoacoustic, and photothermal properties of BP, in addition to the therapeutic aspects previously described, some optimal diagnostic aspects can be simultaneously obtained [7].

Bone regeneration is fundamental in the field of tissue engineering, and many studies have focused on technologies such as 3D bioprinting for the realization of increasingly precise and biocompatible scaffolds. In 2014, Inzana et al. focused their studies on the production of calcium phosphate scaffolds using low-temperature 3D printing. These 3D scaffolds provided excellent results in terms of cytocompatibility and osteoconductive, paving the way for the composition of bio link with substances that are present in bone tissue. BP is present, as previously mentioned, in very low percentages in bones; this fact can be exploited in the composition of bio links with other substances that are favorable to the osteoconduction [35]. Recently, researchers are trying to combine the proliferation and regeneration of damaged or surgically removed tissues. In bone cancer, the challenge is to promote regeneration around the prosthetic device, ensuring the osteogenic and antibacterial properties of the implant surface. Bone regeneration is often supported by scaffolds, made from both organic and inorganic materials that mimic the extracellular matrix of bone. In this context, BP nanosheets contain phosphorus, which is already naturally present in bone. Yang et al. focused their studies on the production of scaffolds made from BP nanosheets combined with BioGlass (BG) by 3D printing. Bio-printing makes it possible to produce scaffolds with complex shapes, sizes, and compositions [36]. In the therapeutic protocol against osteosarcoma, 3D printing with biomaterials doped or coated with BP could be a useful strategy to improve the therapeutic effects. The experimental scaffolds made by Yang et al. were designed with a reticular trauma, similarly to the medullary bone, to promote and improve cell adhesion and colonization. These scaffolds were coated with a BP nanosheet (200–400 nm) that was shown to bind the scaffold structure safely and strongly. The coated scaffolds were tested in vitro; they showed an exceptional ability to improve bone formation, after an improved cell proliferation on their surface, probably due to the peculiar scaffold geometry. Further in vivo studies on a mice model affected by osteosarcoma revealed that the BP-BG scaffolds worked more effectively on post-oncological bone defects [36]. The BP coating was also applied to hydrogels; specifically, a hydrogel was obtained through photo-reticulation with UV of methacrylamide gelatin (GelMA). GelMA was coated with arginine and poly (ester amide) (U-Arg-PEAs), and BP. The functionalized hydrogel showed improved bone formation. In vitro, the mechanical characteristics of the hydrogel were assessed in the presence of BP immersed in substances that simulate body fluids, obtaining a good response in terms of compression modulus and biodegradability time. Furthermore, in the osteogenic differentiation of stem cells from human dental pulp (hDPSC), BP-based hydrogel improved the proliferation of hDPSCs. BP coating appears to be an ideal environment for the growth of hDPSCs, thus demonstrating a potential use of BP-coated hydrogels in the dental field [9].

The possibility of using 2D materials for biomedicine has led researchers to verify their possible use in the therapeutic, diagnostic field. Among the transition-metal dichalcogenides (TMDs), covalent–organic frameworks (COFs), hexagonal boron nitrides (h-BN), metal-organic frameworks (MOFs), layered double hydroxides (LDHs), Wei Tao et al. focused research on BP multifunctionality for Cancer Theranostics. They specifically considered the PEGylated BP Theranostic delivery platform with three different configurations of agents that are capable of drug delivery, photodetection in bio-imaging, and targeting during the photothermal therapy [7]. In vivo tests on mice with platforms composed of nanosheets of BP for DOX therapy were performed, confirming that BP has a greater drug loading capacity than other 2D materials, i.e., MoS_2,_ and graphene. It also responds more quickly to laser radiation with immediate drug release, and shows good photostability and biocompatibility within the body [37]. In photothermal therapy, photothermal conversion and biocompatibility are essential factors, but mechanical performance also plays an important role. PVA hydrogels compounded with BP nanosheets modified with polydopamine, pBP, through freezing and thawing, showed peculiarities in mechanical performance. pBP shows good cellular interactions and effective response to controlled NIR radiation, which is able to dissolve the pBP envelope and release the drug [38]. Currently, nanomedicine drug administration is one of the fundamental areas on which to concentrate studies, and BP appears to be a pioneer. DDS (drug delivery system) are among the most promising techniques in cancer therapy, and BP within hydrogel structures, as seen, provides good results. This feature is interesting for researchers because it is possible to control the biodegradability of the BP envelope based on its composition with the hydrogel and the transmitted NIR radiation. This would make it possible to act more precisely on the area under study [39]. The composition and structural conformation of hydrogels also favor or inhibit the properties of BP. One of the solutions to the easy degradation and oxidation of BP is the use of a hydrogel based on BP nanosheets and cellulose (BPNS). The 3D structure presents nanometric irregularities and pores that yield greater stability, flexibility and effective response to PTT, even in in vivo experiments [40]. Researchers taking advantage of the liquid-base synthesis technique made three different samples of nanosheets, i.e., small S-BP, medium M-BP, and large L-BP. They reported four different types of behavior based on the size of the sample. This suggests that depending on the type of field of application (photothermal therapy, bio-imaging, drug-delivery), BP nanosheets must be correctly sized to be exploited to the fullest of their potential [41]. BP also works as a coating for electrodes or biofilms for bone implants and wearable devices. For example, Xiong J et al. proposed the realization of a tactile triboelectric nanogenerator to be worn on the skin, which is capable of accumulating mechanical energy and transforming it into power [42]. This voltage can be used as a precise self-supply device and for monitoring vital real-time parameters, for the input of mechatronic prostheses and drug release.

## 6. Conclusions

Graphene was discovered several years ago. Since then, it has attracted the attention of researchers and the media throughout the world thanks to the promising properties of 2D nanosheets. The limits of graphene are mainly related to its low biodegradability in vivo and its higher cytotoxicity, compared to BP and other 2D materials. In contrast, the literature has reported that BP has lower cytotoxicity and better degradation rates in vivo, also showing low release rates of nanoparticles in the human body. BP has been demonstrated to serve effectively as a biomedical material, a sensor, an aid in drug release and in most different diagnostic applications. The good stability of its structure and its high ability to link with other biomaterials make it an ideal component in multilayered smart functional materials. Currently, the applications of BP nanosheets in bone tissue engineering are the most promising, both as a scaffold and as a coating on the surfaces of prosthetics to improve the osteoinductive and antibiotic properties of such devices. Nevertheless, the future experimental developments of this novel nanomaterial may lead to several new exciting challenges, particularly with regard to theranostic applications in the field of medical oncology.
